# Intraindividual, intraspecific, and interspecific variation shapes natural selection and its detection in two convergently-evolved lizard species

**DOI:** 10.1371/journal.pone.0326443

**Published:** 2025-08-04

**Authors:** Simone Des Roches, Max R. Lambert, Michaela S. Brinkmeyer, Jacqueline M. Howells, Andy Dettinger, Erica Bree Rosenblum

**Affiliations:** 1 Science Division, Habitat Program, Washington Department of Fish & Wildlife, Olympia, Washington, United States of America; 2 School of Aquatic and Fishery Sciences, University of Washington, Seattle Washington, United States of America; 3 Department of Environmental Science, Policy, and Management, UC Berkeley, Berkeley, California, United States of America; 4 The Nature Conservancy in Washington, Seattle, Washington, United States of America; 5 Department of Biological Sciences, Boise State University, Boise, Idaho, United States of America; 6 Division of Surgical Research, Department of Surgery, Rhode Island Hospital, Providence, Rhode Island, United States of America; 7 Graduate Program in Pathobiology, Brown University, Providence, Rhode Island, United States of America; University of Mississippi, BRAZIL

## Abstract

Much of our understanding of how natural selection operates comes from studies of highly heritable traits presumed to vary little within individuals. Here we show that intraindividual (within-individual) phenotypic variation is an important source of intraspecific variation, shaping both natural selection and its detection in wild, open populations. We employed a multi-year capture-mark-recapture (CMR) study of two lizard species (*Sceloporus cowlesi* and *Holbrookia maculata*) at the ecotone between the white gypsum dunes at White Sands National Park and the surrounding dark Chihuahuan desert soils. Unlike many CMR studies examining selection on morphology, we measured individuals’ traits at each capture. We found that our inferences into which traits were under selection depended on which measurement instance we used (first, last, or median measurement of all measurements of a given trait), and, therefore, the degree of intraindividual variation within each trait. We present a contingency analysis to facilitate assessing when traits are under selection, when they are not, and when intraindividual variation complicates these inferences. Beyond these conceptual advances, our work has implications for the White Sands system, a model system for repeated evolution. In particular, both lizard species experience different selection regimes within the same ecotonal habitat, despite both showing convergent evolution in dorsal blanching on White Sands.

## Introduction

Intraindividual phenotypic trait variation – in particular, morphological variation within individuals through time – is rarely incorporated in research on contemporary natural selection. Researchers studying wild populations typically evaluate selection on phenotypes based on measurements taken at a single time point and on morphological traits that are assumed or demonstrated to be fixed [1–3]. As a result, studies on selection on morphology in wild, open populations typically do not incorporate the effects of growth, condition, and plasticity into their analysis or interpretation. Theoretical frameworks have increasingly incorporated individual variation and repeatability in life history [4], movement [5], behavior and personality [6], and even morphology [7] into natural selection studies. Yet, these frameworks are primarily tested in wild populations with high recapture rates that experience minimal migration [8]. To our knowledge, the measurement of individual variation in morphological traits has yet to be incorporated into survival by trait capture-mark-recapture (CMR) studies on open populations with imperfect detection. Instead, researchers often mitigate noise within individuals by confirming low variation through repeated measures [2,9], or noting high trait heritability [3]. When researchers *do* repeatedly measure variable traits throughout the duration of the study, they often use summary statistics (for example, an average of multiple measurements) in selection models [2,9]. However, intraindividual variation may account for a considerable proportion of among-individual phenotypic variation for traits within (i.e., intraspecific variation) and even across species [10].

Here we took into account intraindividual variation while studying contemporary natural selection on a suite of anatomical and color traits of two lizard species – the Southwestern Fence Lizard (*Sceloporus cowlesi*) and Lesser Earless Lizard (*Holbrookia maculata)* – inhabiting the same transitional ecotone habitat between the gypsum dunes at White Sands National Park and the surrounding dark Chihuahuan desert. The White Sands system is an ideal natural laboratory for studying rapid convergent adaptation: the fence lizard and earless lizard are two of just three lizard species that have convergently evolved blanched coloration on the geologically-young dunes over the past several thousand years [11–13]. Convergence in color in the three species has been studied extensively, including from a molecular perspective [12]. However they also show convergence in multiple ecological traits with unknown heritability, including limb length and trophic head morphology [13–16]. We chose to focus on lizards inhabiting the ecotone between the gypsum dunes and surrounding dark soil desert because (1) this area incorporates environmental characteristics of both adjacent habitats (i.e., white gypsum substrate but with more vegetation, predators, and competitors than in the center of White Sands [11,17]; (2) the two focal species have documented differences in phenotypic plasticity, habitat use, and gene flow across the ecotone, suggesting different levels of intraspecific variation [11,13–16]. Consequently, we expected selection to be strong on the ecotone for *S. cowlesi* because it shows appreciable dorsal color and anatomical variation, including a high frequency of darker phenotypes that would be hypothetically maladaptive on the light gypsum substrate [11,18]. We expected selection to be less strong on *H. maculata*, which show lower trait variation and less migration across the ecotone.

We performed a five-year capture-mark-recapture (CMR) study on *S. cowlesi* and *H. maculata* to determine which traits were under contemporary selection at the ecotone [18]. Recent advances have allowed CMR studies to expand beyond estimating population demographics [19] to evaluate contemporary selection in open animal populations [20–26]. These advances allow estimation of relationships between individuals’ traits and survival by applying Bayesian hierarchical modelling (i.e., Cormak-Jolly-Seber, “CJS” models) using uniquely-marked individuals. Although the CJS approach is increasingly advocated for estimating survival by phenotype [22,27–30], to date, it is still a relatively uncommon method for measuring ongoing natural selection [but see 31,32]. Further, because CJS models compare survival across individuals with different trait values, they necessarily focus on variation at the intraspecific level. As such, like many natural selection studies, CJS models using CMR data typically do not incorporate intraindividual variation, nor do they compare survivorship between species. Rather, they use a single species to incorporate individual trait measurements taken at a single point in time, assuming relatively static trait variation throughout individuals’ lifetimes [9].

In our study, we attempted to expand the potential of CMR studies with CJS models to evaluate selection on multiple traits while incorporating variation at the individual, intraspecific, and interspecific levels. By doing so, we explore shared and divergent selection regimes in two species, while acknowledging the complicating role of intraindividual variation on the process and detection of selection on multiple traits. Here we describe our discovery of how this intraindividual variation influenced our analysis of contemporary selection. We further provide a framework for future research and highlight challenges for the field going forward.

## Methods

### Study system

We performed a five-year capture-mark-recapture (CMR) study using Cormack-Jolly-Seber (CJS) models to assess differences and similarities in natural selection on phenotype (color and anatomical traits) in two lizard species – *Sceloporus cowlesi* (the Southwestern Fence Lizard) and *Holbrookia maculata* (the Lesser Earless Lizard) –exhibiting convergent evolution in a novel habitat. Our study was located on the ecotone of White Sands, New Mexico, a geologically young ecosystem composed of 650 km^2^ of gypsum sands, which have been deposited over the last 2000–7000 years [33,34]. White Sands’ gypsum sand dunes sharply contrast with the surrounding dark brown alluvial, loamy soils of the Chihuahuan Desert. Multiple species have rapidly adapted to White Sands and three lizard species in particular exhibit substantial phenotypic differences from their conspecific counterparts inhabiting the surrounding dark soil desert [13–15,35]. The most notable difference between lizards in the two habitats is in dorsal coloration: populations of all three species are blanched on White Sands and dark brown in the rest of the Chihuahuan Desert. Coloration in these lizards has a heritable component and is an ostensible adaptation for crypsis on the gypsum substrate [11,13,36–39]. White Sands lizards also display varying degrees of anatomical differentiation from their darker counterparts, having longer limbs thus faster sprint speed [14], and larger heads associated with a diet including larger, harder invertebrates [15].

### Sampling

We focused our study on *H. maculata* and *S. cowlesi* inhabiting the south-eastern ecotone of White Sands, New Mexico. We exhaustively captured all lizards observed in a single area of nearly 10.7 ha, which extended approximately 200 m from the ecotone westward into White Sands (UTM: 13S 388460–388640 mE, 3625250–3725780 mN). Both species were primarily associated with vegetation in large ‘interdune’ areas, which were enclosed by 3–10-m-high dunes. We captured individuals by lasso or by hand between 07:00 and 15:00 and returned all lizards 1–2 days later during daylight hours at the precise location of capture after measuring, identifying, and uniquely marking them. No animal sacrifice, anesthesia, or analgesia were necessary as part of this study. All live animal work was conducted with relevant Animal Care and Use Committee permits (University of Idaho, protocol number 2010–48 and University of California, Berkeley protocol number R347). Access to sites was provided by New Mexico Department of Fish and Game and by the White Sands National Monument.

We sampled *H. maculata* from 2011 to 2014 and *S. cowlesi* from 2012 to 2015. We sampled at the beginning of the activity season each year between May and June. For the first sample year for each species (2011 for *H. maculata* and 2012 for *S. cowlesi*) we additionally sampled at the end of the activity season (August). We maintained consistent sampling effort each year by recording person hours surveying each interdune area and adjusting based on need. We terminated capturing in an interdune area once we had not caught any new (that year) lizards for one day (approximately 5 hours) with four samplers. We measured anatomical and color traits of each individual in the lab at each capture event.

We uniquely marked individual lizards with manual injection visual implant elastomer (Northwest Marine Technology Inc.), which has been used effectively for reptiles in other CMR studies [40]. We used up to six different fluorescent colors in four locations on lizards’ ventral surface and upper thigh. We used a combination of photographs and ventral patterning to further help identify individuals with missing or faint tags.

### Measuring anatomy

We measured anatomical traits directly from live lizards and digitally from ventral body scans. For consistency, the same person (SD) performed all live and digital measurements on all individuals across years. We measured head depth (highest part of the skull, midway above eye to below jaw), head length (tip of snout to behind lower jaw), and head width (at widest point), as well as pelvic width to the nearest 0.1 mm on individuals using hand-held callipers. We measured lizard weight to the nearest 0.1 g from lizards suspended in a plastic bag affixed to a Pesola spring scale [41]. At this time, we also took ventral scans of individuals using a flatbed scanner, holding them flat with light pressure and left limbs and tail extended straight from the body. From scans, we digitally measured snout-vent-length (SVL), interlimb length (from posterior insertion of forelimb and anterior insertion of hindlimb), fore and hindlimb length (from shoulder to tip of longest toe). Morphological measurements from digital images obtained using flatbed scanners have been used for various morphological measurements in lizards [42,43]. We measured a subset of 50 lizards multiple times to ensure consistency while in the field. We performed all subsequent analyses on morphological traits adjusted for SVL (i.e., using the residuals of the linear model for the regression of trait measurement against SVL), including lizard condition, which was calculated from the regression of weight on SVL. To ensure the measurer was consistent, we performed a repeatability analysis for four digitally-measured anatomical traits (SVL, interlimb, forelimb, hindlimb lengths) from ventral scans on a random subset of 12 individuals of each species (all R > 0.94, see Supplement).

### Measuring color

We measured lizard color from digital photographs taken under standard conditions [44]. We took photos with a Nikon D5100 with a 50-mm lens (shutter speed: 1/160, 100 ISO) after lizards spent 2 minutes in a warmed basking tank. We photographed lizards on white poster board with a white, grey, and dark grey color standard (Adorama QPcard 101) and ruler.

We measured the CIE (International Commission on Illumination) L*a*b color space from digital photographs in Adobe Photoshop CS7. This color space is perceptually uniform such that numerical change corresponds to similarly perceived change in color [45]. The L*a*b color space allows representation of an infinite number of colors in three-dimensional real number space [46] and has been used extensively to measure color in vertebrates, including reptiles [47] as it is an objective measure of visible color for diurnal vertebrates in terrestrial environments [48]. The L* value (corresponding to relative lightness) ranges from 0 (completely black) to 100 (completely white), the a* corresponds to green-red opponent colors (green = negative values, red = positive values), and the b* corresponds to blue-yellow opponent colors (blue = negative values, yellow = positive values).

To measure color, we first set dark and light color standards with the scale bar in each photograph using the eyedropper and curves function in Photoshop. With the Rectangular Marquee tool, we used the photographed ruler to determine the number of pixels corresponding to 1 mm. We then blurred and averaged the color in a fixed 4 mm by 4 mm square on each of the dorsal and dorsolateral surface of each lizard, approximately halfway down the body. Finally, we measured L*a*b color using the eyedropper tool.

### Measuring detection & survival

To estimate detection and survival probability, we used open population capture–recapture [19,49] implemented in the R package “marked” to fit CJS models using maximum likelihood estimation (version 1.2.6, [50]). CJS models [19,51] model recaptures only, conditioning on their first capture. The model provides estimates of the probability of detecting an individual during sampling (detection probability = *p*) and the probability of surviving from time *t* to *t* + 1, (survival probability = *φ*). Survival probability was logit-transformed and modelled as a function of covariates [19], which included anatomical and color measurements. We used the analysis protocol described in Laake et al. [50], modelling *p* as constant across time and individuals and *φ* as constant across time.

We used Akaike Information Criteria (AIC) to compare a null intercept-only model against univariate models for each of six color traits (dorsal and dorsolateral L*a*b) and nine anatomical traits (SVL, body condition, and SVL-adjusted pelvic width, interlimb length, head length, depth, width, forelimb and hindlimb length). We ran separate model comparisons on color and anatomical traits and on the first, median, and last trait measurement instance (for individuals captured more than once – see explanation below). Importantly, because we captured new individuals each year, the first measurement was taken in different years for different individuals. Therefore, there is no reason to expect a correlation between measurement instance and year. We considered first, median and last models to be consistent if they all had ΔAIC values less than the default (no trait) model.

### Measuring variability

We recognized that variation within individuals across years (intraindividual variation) might underlie incongruence among selection analyses performed on the first, median, or last measurement instance of the same trait in different years. To identify traits showing selection analysis incongruence due to intraindividual variation, we performed linear models (using the ‘lm’ function) on each trait for individuals with multiple measurements across years (i.e., with at least one recapture) using individual lizard identity as a predictor variable. We used F-statistics (ratio of intraspecific to intraindividual variation) from these models to infer the relative degree to which intraindividual versus interindividual variation explained overall phenotypic variation. In all cases higher F-statistics reflected a higher proportion of among-individual variation than intraindividual variation (i.e., F > 1). Higher R^2^ values (i.e., R^2^ > 0.5 meaning over 50% of the trait variability was attributable to variation across individuals) provided additional support for the strength of among-individual differences in explaining phenotypic variation for a particular trait.

By combining these test statistics with ΔAIC statistics from selection models on the first, median, and last measurement instance of each trait, we inferred our degree of certainty in assessing selection (Table 1). For instance, we confidently concluded a trait was experiencing directional selection if it had a high F-statistic and R^2^ (i.e., high among versus within-individual variation) and consistently negative ΔAIC values for models on first, median, and last measurement instance. In contrast, a trait with a high F-statistic and R^2^ and positive ΔAICs is unlikely experiencing strong selection. For traits with positive or inconsistent ΔAIC values across measurements, a low F-statistic and R^2^ values suggested high intraindividual variation likely leading to spurious conclusions about selection for these traits. In these cases, making inferences into selection is challenging because high intraindividual variation either interferes with selection analyses or makes it challenging for the trait to respond to selection.

**Table 1:** Contingency table for evaluating confidence in whether a trait was or was not experiencing selection given F-statistics and R^2^ values from ANOVA comparing intraspecific to intraindividual variation and ΔAIC on models using first, median, or last measurement instance compared to intercept only model.

	Higher intraindividual variation: Low F-statistic & R^2^ > 0.5	Lower intraindividual variation: High F-statistic & R^2^ > 0.5
**Weaker evidence of directional selection:** ΔAIC > 0 for all or some measurements across time	High intraindividual variation obscures or inhibits inference of selection (e.g., *S. cowlesi* pelvic width, hindlimb length; *H. maculata* dorsolateral lightness, pelvic width, head length/depth)	Evidence trait is not currently under selection (e.g., *S. cowlesi* dorsal and dorsolateral lightness/blue-yellow color, dorsolateral green-red color, SVL, head length/width, forelimb length; *H. maculata* dorsal lightness, dorsal and dorsolateral green-red/blue-yellow color, interlimb/ forelimb/hindlimb length)
**Stronger evidence of directional selection:** ΔAIC < 0 for all measurements across time	High intraindividual variation means possibly spurious conclusion that a trait is currently under selection depending on when measurement is taken. (e.g., *S. cowlesi* pelvic width, head length; *H. maculata* SVL, head length)	Evidence trait is currently under selection (e.g., *S. cowlesi* dorsal green-red color, interlimb length, head depth; *H. maculata* head width)

## Results

Our five-year capture-mark-recapture (CMR) study revealed the interconnected roles of intraindividual and intraspecific trait variation for ongoing natural selection on two syntopic lizard species (*Sceloporus cowles*i and *Holbrookia maculata*) at the White Sands ecotone. In our study, the extent of both intraindividual (within-individual) and intraspecific (among-individual) variation differed substantially among traits and between the two species. Certain traits in both species, for example, were more likely to show both appreciable intraindividual and intraspecific variation, such as body condition and pelvic width, whereas other traits showed more intraspecific relative to intraindiviudal variation, such as dorsal lightness and forelimb length. Our analyses revealed that intraindividual trait variation is central to detecting and understanding contemporary natural selection in White Sands and is likely an important, though often unaccounted for, consideration in other systems (but see [7,8]). Specifically, intraindividual variation both complicated our ability to infer which traits experience selection and also revealed the traits that are likely undergoing the most consistent and strongest selection.

### Demographics and survival

Population density, sex ratio, survival probability, and recapture probability differed between the two species surveyed at the White Sands ecotone (Table 1 in S1 File). Jolly-Seber models estimated population density for *S. cowlesi* (13.1 individuals/ha) to be over double that of *H. maculata* (6.4 individuals/ha) and a balanced sex ratio in both species. CJS models showed that survival probability was more than 20% lower for *S. cowlesi* at 0.45 + /- 0.55 (mean + /- standard error) compared to *H. maculata* at 0.68 + /- 0.56 but similar recapture probabilities for both (*S. cowlesi*: 0.55 + /- 0.58; *H. maculata*: 0.57 + /- 0.57).

### Survival by phenotype: color and anatomical traits

**Fig 1:**
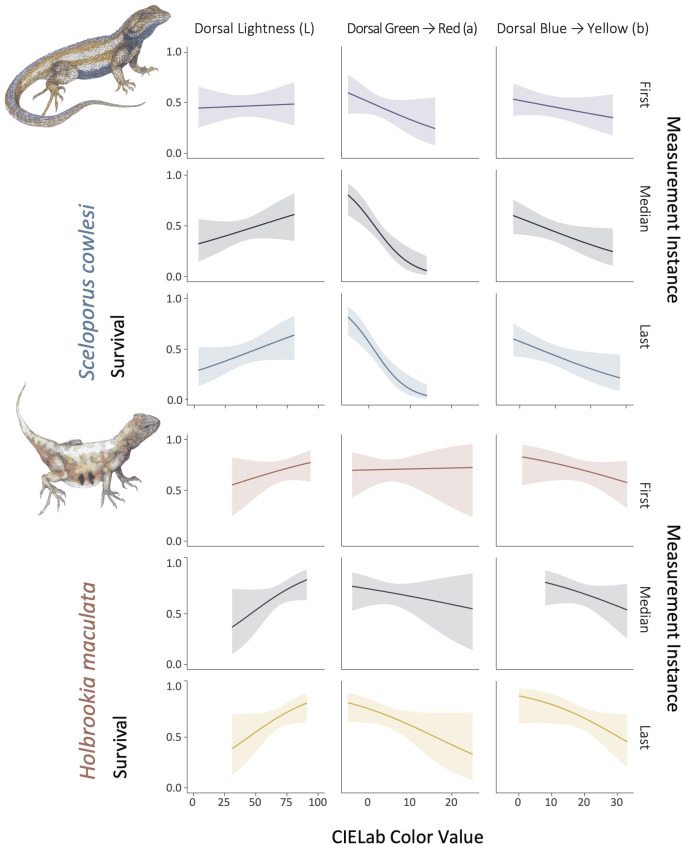
Cormack-Jolly-Seber model outputs displaying the relationship between survival and three axes of CIELab color space of the dorsal surface for *Sceloporus cowlesi* (top) and *Holbrookia maculata* (bottom). From left to right: lightness (L), green to red (a), and blue to yellow (b). Models are fit using the first (purple = *S. cowlesi*, orange = *H. maculata*), median (black), and last (blue = *S. cowlesi*, yellow = *H. maculata*) measurement instance for repeatedly-captured individuals. Similarity among curves for the first, median, and last measurement instance is partly attributable to lower intraindividual variation in measurements among years for recaptured individuals, and indicates higher reliability of estimates.

**Fig 2:**
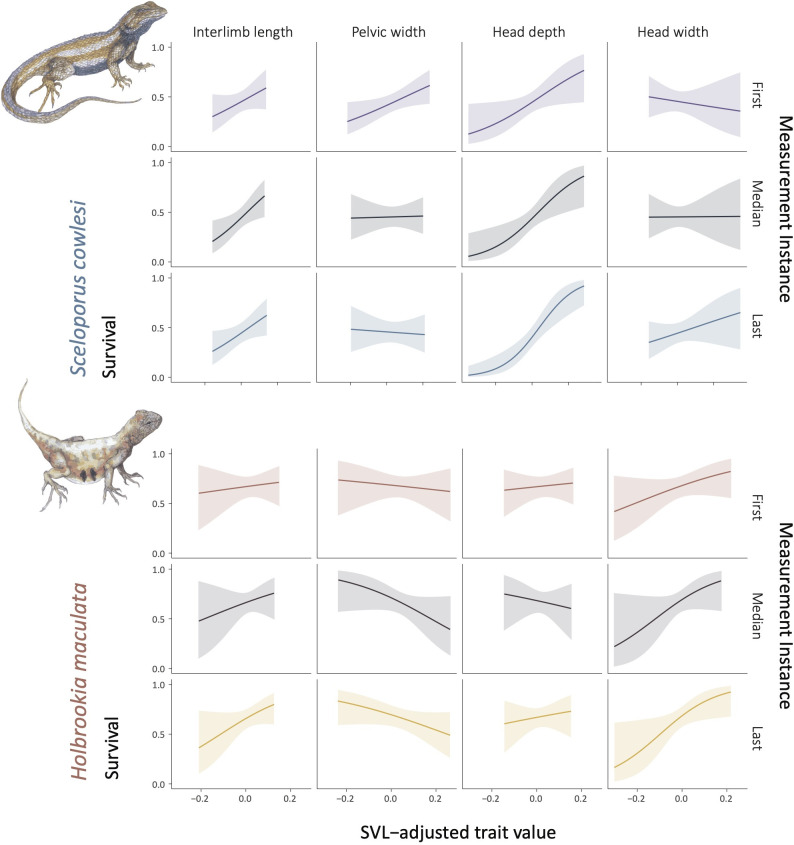
Cormack-Jolly-Seber model outputs displaying the relationship between survival and four measures of size-adjusted body shape for *Sceloporus cowlesi* (top) and *Holbrookia maculata* (bottom). From left to right: interlimb length, pelvic width, head depth, and head width. Models are fit using the first (purple = *S. cowlesi*, orange = *H. maculata*), median (black), and last (blue = *S. cowlesi*, yellow = *H. maculata*) measurement instance for repeatedly-captured individuals. Similarity among curves for the first, median, and last measurement instance is partly attributable to lower intraindividual variation in measurements among years for recaptured individuals, and indicates higher reliability of estimates.

Relationships between survival and phenotype varied with species, trait, and the measurement instances used for recaptured individuals (i.e., measurement at first capture, last capture, or median value across captures). In general, *S. cowlesi* showed both lower survival overall and stronger evidence of a relationship between survival and phenotype than *H. maculata* for both color (green-red dorsal color value, Fig 1, Table 2 in S1 File) and anatomical traits (interlimb length and head depth, Fig 2, Table 3 in S1 File). Whereas there was only evidence for selection on *H. maculata* head width. Models using the last measurement (for individuals with multiple captures) generally demonstrated stronger relationships between survival and phenotype, although it is unclear why, given that this last measurement occurred in different years for different individuals. Consistent patterns and support for models of survival by trait value using the first, median, and last measurement instance were taken as the best evidence for selection on a particular trait.

There was weak support for selection on dorsal and dorsolateral color in both *S. cowlesi* and *H. maculata*. Though there was a consistent positive association between survival and dorsal lightness (Fig 1, Table 2 in S1 File) for both species and regardless of measurement instance (first, median, last), these models did not outperform the null (intercept only) model (ΔAIC < 2). Models showed support for survival based on dorsal green-red values for *S. cowlesi*, with greener individuals having slightly higher survival (Fig 1, Table 2 in S1 File). Though trends were similar for *H. maculata* color, no trait model consistently outperformed the null model.

**Fig 3:**
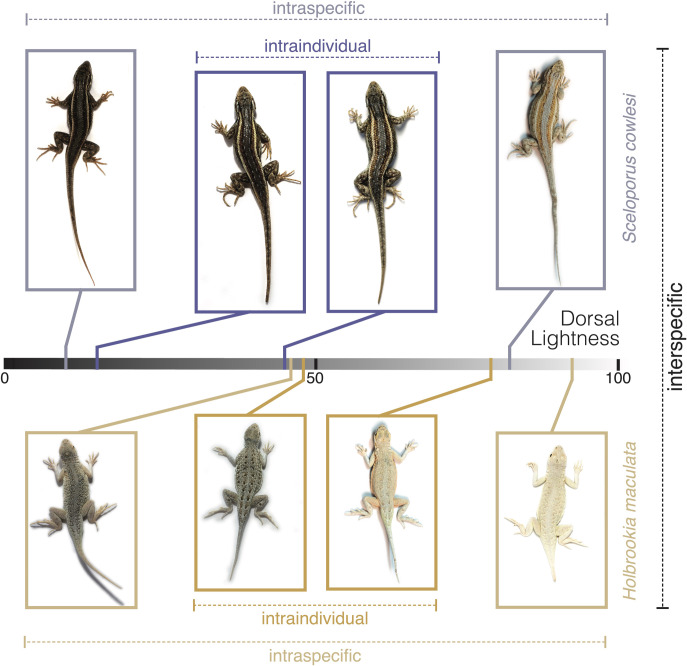
Representative photographs used for CIELab color analysis for the two species, *Sceloporus cowlesi* and *Holbrookia maculata* showing examples of interspecific (between the two species), intraspecific (within each species), and intraindividual (within an individual across sample events) variation in dorsal lightness from 0 (black) to 100 (white).

There was some support for selection on anatomical traits in both *S. cowlesi* and *H. maculata*. Survival was consistently positively associated with size-adjusted interlimb length and head depth for *S. cowlesi* and with head width for *H. maculata* (Fig 3, Table 3 in S1 File). Positive associations between survival and adjusted pelvic width (Fig 3) and head length for *S. cowlesi* and adjusted head length (not displayed) for *H. maculata* outperformed the null model; however, these results varied depending on whether the first, median, or last measurement instance was used. Snout-vent-length was negatively associated with *H. maculata* survival, however, this trait unsurprisingly showed substantial intraindividual variation due to growth, suggesting caution should be taken regarding its interpretation.

### Intraindividual, intraspecific, and interspecific variation

**Fig 4:**
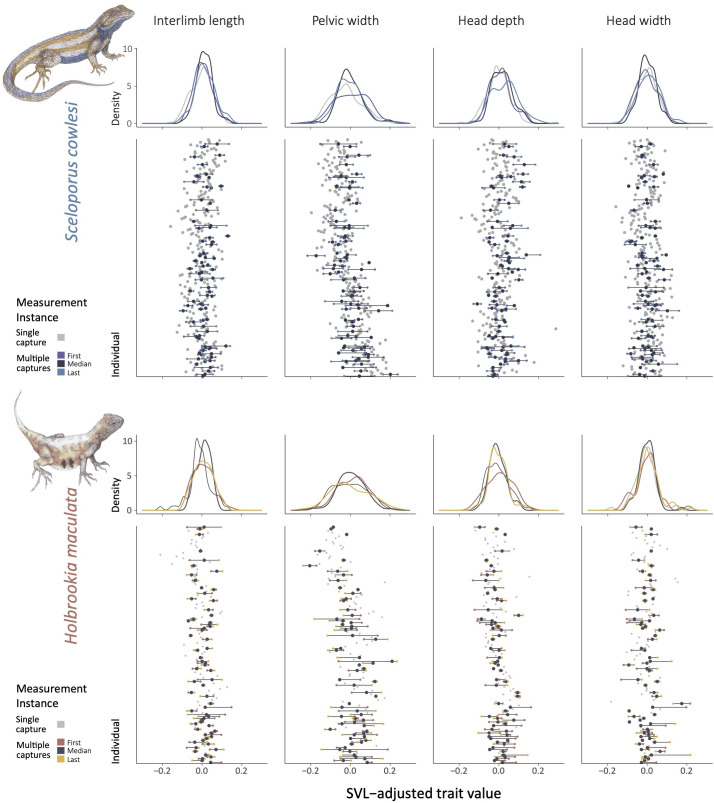
Intraindividual, intraspecific, and interspecific variation in three axes of CIELab color space of the dorsal surface for *Sceloporus cowlesi* (top) and *Holbrookia maculata* (bottom). From left to right: lightness (L), green to red (a), and blue to yellow (b). Density plots demonstrate differences in the color distributions of first (orange), median (black), and last (yellow) measurement instance for individuals with multiple captures, and the only measurement instance (grey) for individuals with a single capture. Lower overlap among curves is an indication of more variation within individuals, whereas flatter curves indicate more even variation among individuals. Point plots display variation within and among individuals directly, and are also colored by first (orange), median (black), and last (yellow) measurement instances for individuals with multiple captures and single captures (grey).

**Fig 5:**
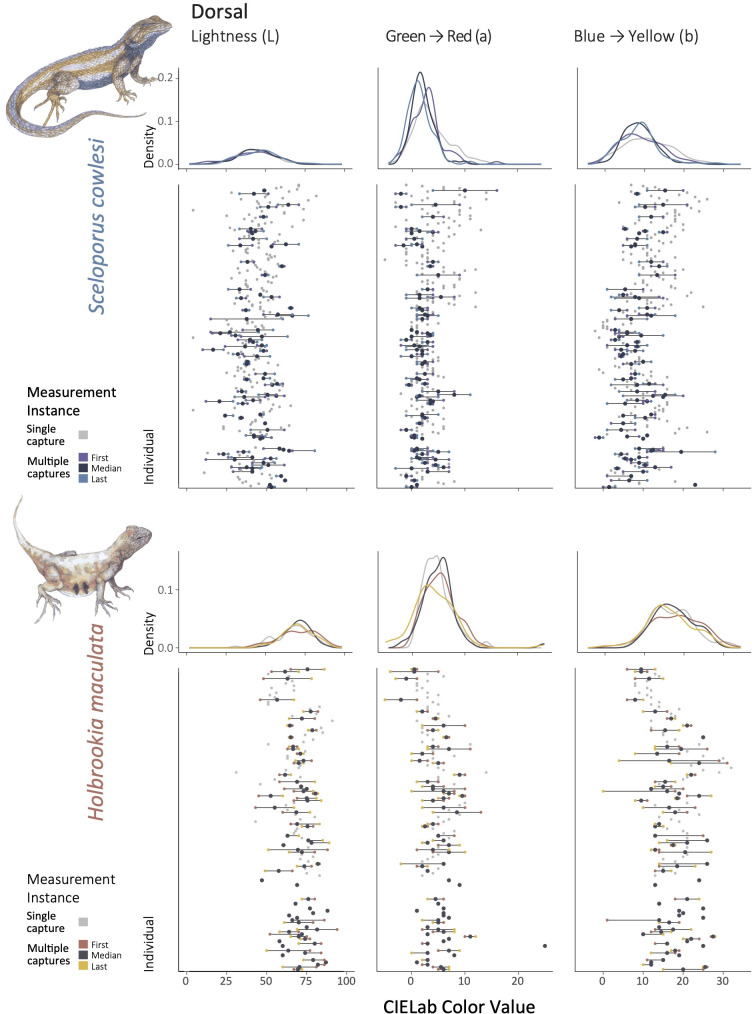
Intraindividual, intraspecific, and interspecific variation in four measures of size-adjusted body shape for *Sceloporus cowlesi* (top) and *Holbrookia maculata* (bottom). From left to right: interlimb length, pelvic width, head depth, and head width. Density plots demonstrate differences in distributions of first (orange), median (black), and last (yellow) measurement instances for individuals with multiple captures, and the only measurement (grey) for individuals with a single capture. Lower overlap among curves is an indication of more variation within individuals, whereas flatter curves indicate more even variation among individuals. Point plots display variation within and among individuals directly, and are also colored by first (orange), median (black), and last (yellow) measurement instances for individuals with multiple captures and single captures (grey).

The relative ratio of intraindividual (within individuals) to interspecific (across individuals within species) variation differed among color traits and between the two species (Figs 4, 5, Table 2 in S1 File). For *S. cowlesi*, the degree of intraspecific compared to intraindividual variation was relatively high for all color traits (F > 4.0, R^2^ > 0.50), indicating that intraspecific variation was responsible for over half of the observable variability. This was especially true for dorsal and dorsolateral lightness, which had a relatively low degree of intraindividual variation (F > 7.0, R^2^ ~ 0.70). For *H. maculata*, intraspecific variation relative intraindividual variation was also high for color traits, with the exception of dorsolateral lightness, which showed considerable variation within individuals (F = 2.5, R^2^ = 0.33).

The two species differed considerably in which anatomical traits showed more intraindividual versus intraspecific variation (S3 Table in S1 File, Fig 5). For *S. cowlesi*, the trait showing the lowest degree of intraspecific compared to intraindividual variation was body condition (F < 3.0, R^2^ < 0.40), whereas for *H. maculata* it was body size (F < 2.0, R^2^ < 3.0). Both species showed higher intraspecific relative to intraindividual variation in interlimb length, but this was reversed for pelvic width. For *S. cowlesi,* head length and depth showed more relative intraspecific variation (F > 7.0, R^2^ > 0.65), whereas for *H. maculata*, there was more relative intraindividual variation in these traits (F < 4.0, R^2^ < 0.50). *S. cowlesi* forelimb length had higher relative intraspecific variation than hindlimb length, whereas in *H. maculata*, both had higher intraspecific variation.

## Discussion

Natural selection acts on intraspecific variation within populations, a large part of which can be variation within individuals. Although studies on behavior and life history have examined the fitness implications for intraindividual variation [6,7,52,53], morphological traits are largely (often implicitly) assumed to be fixed in natural selection studies, varying across individuals within a population more than within individuals over time (e.g., [1–3]). This has considerable implications for how we infer selection in wild populations, especially in CMR studies. For example, certain traits that could be more closely tied to physiological condition and growth, such as body condition and pelvic width, show considerable intraindividual variation in both species, thus complicating the inference of ongoing natural selection on these particular traits. Although dorsal and dorsolateral lightness is largely heritable in both White Sands lizard species studied here [11], intraindividual variation is relatively higher for *H. maculata*, which has been shown to exhibit higher physiological and lower developmental plasticity [54]. This means first that natural selection is effectively acting on a “moving target” (*i.e.*, a trait that plastically changes in response to natural selection) and, second, that our ability to detect this selection is contingent on when the trait is measured [8].

### Intraindividual variation in capture-mark-recapture studies

Capture-mark-recapture studies often assume that focal traits are constant throughout the study’s duration or individuals’ lifetime (e.g., [1–3]). As such, researchers commonly focus on traits shown to be significantly heritable (e.g., red knot bill size [2], great tit bow tie size [55]) or are assumed as such (e.g., adder color polymorphism [32]). Though heritability is necessary for evolution by natural selection, heritable traits are not necessarily static through an individual’s lifetime [7]. In particular, traits can vary with growth or condition, and even when size-adjusted, measurements often do not scale isometrically through development [56]. Studies often successfully mitigate this issue by using individuals of a similar size and age class to minimize ontogenetic effects [57]. Still, traits can vary unpredictably within individuals across time, even when age and size class are accounted for [9]. Further, the strength of selection can vary on a trait depending on when during selection that trait is measured [2].

Findings from CMR studies naturally increase in accuracy with the number of capture events, which provide increased probability of correctly evaluating detection [28]. However, increased time in between captures necessarily means more opportunity for growth and changes in condition, and therefore higher intraindividual trait variation. Therefore, for many traits, a single measurement is likely inadequate to characterize the whole of an individual’s trait value (both absolute and size-adjusted) and thus its response to selection [7]. Choosing less transmutable, strongly heritable traits mitigates some of this variation. Still, all phenotypic traits are subject to some variability through time and natural selection acts on these traits regardless of this variability [10]. As such there is a great need for trait repeatability to be measured, acknowledged, and quantitatively accounted for in CMR design and models.

### Intraindividual variation complicates the detection of selection

In our White Sands study, we were more confident about the role of natural selection when there was lower trait variation within individuals (i.e., high F-statistic, R^2^ > 0.5). In general, low intraindividual variation in a trait meant that CMR provided strong evidence that it was either likely or not likely under selection. For example, not only did *S. cowlesi* head depth show a lower proportion of variation within- versus among-individuals, but models also showed larger heads were consistently selected for, regardless of whether the first, median, or last measurement instance was used for recaptured individuals. On the other hand, *H. maculata* hindlimb length and *S. cowlesi* dorsal lightness both showed lower within- versus among- individual variation, but models showed little evidence of directional selection regardless of measurement used. This reinforces how identifying when a trait is not under selection is just as important as identifying when it is under selection. Although an absence of evidence is not evidence for an absence of effect, our contingency assessment framework (Table 1) provides guidance for identifying traits that researchers can be generally confident are likely or unlikely experiencing contemporary selection.

Contrastingly, high intraindividual variation in a trait meant that CMR models could not provide reliable evidence of selection. This occurred either because conclusions about selection on a trait depended on the timing of measurement or selection did not act consistently on traits that changed through time (e.g., *H. maculata* dorsolateral lightness). For example, pelvic width of both species showed substantial variation within- versus among- individuals. Correspondingly, models showed different relationships between trait values and survival depending on whether we used the first, median, or last measurement instance. For *H. maculata* head depth, which also varied considerably within individuals, models showed no evidence of selection regardless of measurement used, potentially indicating that the trait varies too much through an individual’s lifetime to be under consistent, perceptible selection.

### Using multiple traits in multiple species for capture-mark-recapture studies

One strength of measuring survival as it relates to multiple traits simultaneously is that it is theoretically possible to test relationships and collinearity among such traits. Traits may be genetically, physiologically, or phenotypically linked and they may be selected on together or in opposition [58]. Though studies have explored trait relationships through pleiotropy, genetic linkage, and/or hitchhiking (see review in [59]), we are unaware of any method that tests for these mechanisms using CMR data, while incorporating imperfect detection. Further, though multivariate methods have been explored in the CMR literature [60], we are not familiar with analyses that explicitly model the relationship between survival and multiple traits simultaneously, let alone while integrating intraindividual variation. Generating new analytical tools that incorporate intraindividual variation in multiple interacting traits using CMR data is a monumental task, but one which will contribute to a more comprehensive understanding of natural selection in the wild.

Studying multiple traits and multiple species simultaneously did allow us to compare the extent of variation at the intraindividual, intraspecific, and interspecific levels. Such multi-level approaches are rarely used in CMR natural selection studies, which, as discussed above, tend to focus on one-time measurements, but also on only one or a few individual traits in a single species. Measuring multiple traits repeatedly in multiple species provides valuable information about the relative composition of phenotypic variation at different levels and how it differs among species. In particular, this approach enables researchers to answer questions about whether certain traits are more or less variable within individuals compared to other traits and whether this variability is similar (e.g., pelvic width) or different (e.g., fore and hindlimb lengths) between different species. These results demonstrate that the observable intraspecific variation within different species – including for the same trait – is comprised of differing degrees of within- versus among-individual variation, even when species show strong phenotypic convergence and occupy the same habitat.

### Implications for ongoing natural selection on White Sands lizards

By more confidently determining which traits were and were not under selection, we demonstrate contrasting selection regimes on two syntopic species. Though White Sands is an iconic natural laboratory for studying convergent evolution, our analyses suggest that *S. cowlesi* and *H. maculata* may be experiencing distinct selection regimes on different traits at the ecotone. In other words, convergence among White Sands lizards in color and anatomy might reflect that shared historical selection regimes are stronger than contemporary selection. In particular, whereas several traits (e.g., dorsal green-red color, interlimb length, and head depth) were associated with higher survival in *S. cowlesi*, only head width showed a relatively strong signal of selection in *H. maculata*, a trait that could be associated with a diet of larger, harder invertebrates [15,16].

Differences in selection between the two species could reflect their demographic differences and varying distances from local fitness optima [37]. In particular, *H. maculata*, which was less intraspecifically variable across traits, shows limited gene flow across the ecotone, which it likely experiences as an extension of the White Sands habitat [11,13]. As such, the ecotone population is more likely to be closer to their fitness optimum. In contrast, the more intraspecifically variable *S. cowlesi*, which demonstrates substantial gene flow from both habitats, might experience the ecotone as a distinct habitat type across the ecotone [11,13]. Thus, this species is likely farther from a fitness optimum. Despite pronounced evidence for convergent evolution in the past [11–13], these two species show variable patterns of contemporary selection because of their differing demography and ecology in the transitional ecotone habitat.

Natural selection on color – particularly dorsal brightness – has been a central research focus for the White Sands lizards [11–13,36–39]. Though there are stark differences in color between blanched lizards on the gypsum dunes and their darker conspecifics inhabiting the surrounding dark soils, we found only limited evidence of selection for dorsal brightness for both species at the ecotone. This was particularly surprising for *S. cowlesi*, which displays considerable intraspecific color variation, even on the ecotone’s light substrate [11]. Weaker selection on ecotonal *S. cowlesi* could be a reflection of its habitat use: unlike *H. maculata*, which primarily basks and forages on the ground, *S. cowelsi* is usually associated with vegetation, particularly in the dark soil habitat [17] and presumably on the ecotone, potentially reducing the importance of background substrate matching. Further, this ostensibly clear case of convergent evolution on dorsal brightness may reflect historical selection that is no longer as strong, particularly on the ecotone. For example, declining populations of avian predators in New Mexico, such as kestrels and shrikes, might have contributed to reduced selective pressures [61–64]. As such, our study underscores how inferences into selection at one point in time and in one location may not translate into how it operates currently and elsewhere across a landscape, even when perceived selective pressures are similar.

Additional attributes of crypsis, such as other dimensions of color (i.e., green-red and blue-yellow hues) and patterning may be overlooked components of lizard intraspecific variation under selection at White Sands. Apart from dorsal brightness, models consistently showed evidence of selection for “greener” and against “redder” *S. cowlesi* individuals. Although the ecological reason behind this pattern is unclear, photographs suggest that differences on the green-red spectrum might reflect width variation in dorsal stripes, rather than overall dorsal color itself. Further work should expand investigation beyond dorsal brightness, such as variability in other dimensions of color and the contribution of dorsal pattern.

## Conclusions

Our study offers a robust framework for future work on ongoing natural selection in wild, open populations. First, measuring additional traits beyond those which are hypothesized to be under selection can help generate new hypotheses about the selective pressures a population is experiencing. Second, analyzing traits that are ostensibly static throughout an individual’s lifetime can facilitate detecting selection. If the degree of intraindividual variation for a trait is unknown, repeated measurements at different time points can be used to assess confidence in detecting selection on that trait. Third, if possible, conducting studies in environments where populations are expected to be further from their fitness optimum can maximize the chances of detecting strong selection. Fourth, and relatedly, measuring multiple species – particularly species which may differ in their relative locations on their respective fitness landscapes – will provide useful contrasts for inferring the strength of selection and whether syntopic species experience selection similarly. We provide a conceptual contingency assessment for this type of work, however, quantitative advancements that allow explicit integration of repeated phenotypic measures into CMR analyses will greatly facilitate research on natural selection.

Natural selection is a complex historical and ongoing process shaping variation within individuals, populations, and across species. Our work demonstrates that contemporary selection regimes can differ among species, even if their evolutionary histories are convergent for certain traits. Importantly, our study underscores the role that intraindividual variation plays in influencing the process and study of selection.

## Supporting information

S1 File Supporting Repeatability Analysis, Demographic Estimates and Summary Statistics. Anatomical measurement repeatability analysis results figure, demographic summary statistics and estimates (table), and within versus among individual variation in color and anatomy analysis results (2 tables).
(DOCX)
