# Supplementary material for: Intraindividual, intraspecific, and interspecific variation shapes natural selection and its detection in two convergently-evolved lizard species
Source: PLoS One. 2025 Aug 4;20(8):e0326443. doi: 10.1371/journal.pone.0326443 (PMC12321066; doi:10.1371/journal.pone.0326443)
Supplement: S1 File — Anatomical measurement repeatability analysis results figure, demographic summary statistics and estimates (table), and within versus among individual variation in color and anatomy analysis results (2 tables). (DOCX) [file pone.0326443.s001.docx]

**Supporting Information:**

**Intraindividual, intraspecific, and interspecific variation shapes natural selection and its detection in two convergently-evolved lizard species**

**Repeatability Analysis**

We used the R package “rptR” (Stoffel et al 2017) to gauge trait measurement repeatability (R) and therefore reliability from the same measurer. Repeatability is calculated as R = VG/(VG+VR) where VR is variance in repeated measurements (data-level = “rep”), and VG is a combination of intraindividual (“indv”) and intraspecific (“intra”) variance (group-level). To measure VR, we took a subset of 12 unique individuals from each species, which were each recaptured (at least) once. All lizards had been measured by SD between 2011 – 2016 using ImageJ and were measured again by SD in June 2023. Repeatability was always greater than 0.94, demonstrating that variation within individuals across years, and across individuals was much greater than that which could be attributed to measurement error. It was only possible to perform repeatability analysis on traits that were measured from ventral scans.

Stoffel, M. A., Nakagawa, S. and Schielzeth, H. (2017), rptR: repeatability estimation and variance decomposition by generalized linear mixed-effects models. Methods Ecol Evol, 8:1639???1644. doi:10.1111/2041-210X.12797


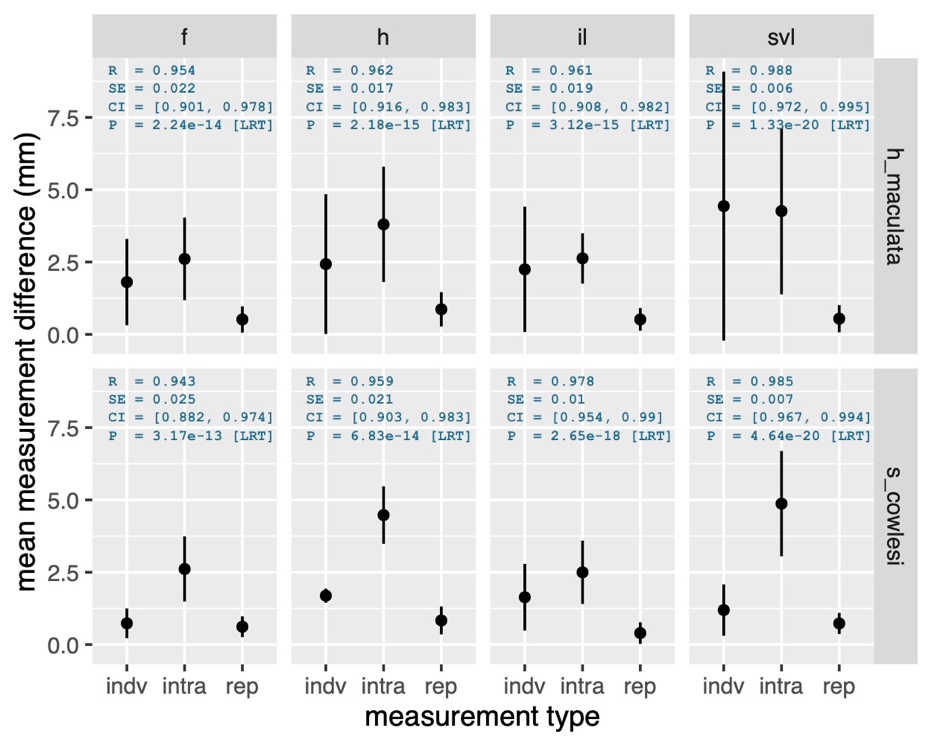


**Fig 1: Anatomical Measurement Repeatability Analysis Results:** Mean difference between two measurements taken on the same photo, by the same person (SD), several years apart (“rep”) versus recaptured individual variation across years (intraindividual variation = “indv”) and across individuals (intraspecifc variation = “intra”). Error bars represent standard deviation of the mean. Outputs of repeatability analysis shown on each plot for each trait (f = forelimb, h = hindlimb, il = interlimb, svl = snout vent length) for each of the two species.

**Table 1: Demographic Summary Statistics and Estimates:** Summary of estimated population size (N), density (N/ha), sex ratio (F:M), survival probability with standard deviation (ɸ ± σ_x_), recapture probability with standard deviation (p ± σ_x_), and mean, and standard deviation (x̅ ± σ_x_) for three dorsal color space traits, and three anatomical traits (adjusted by snout-vent-length: SVL) for the two species, *Sceloporus cowlesi* and *Holbrookia maculata* at the White Sands ecotone. For individuals captured multiple times, the median of all capture measurements was used. Models do not allow the estimation of capture and survival probabilities with fewer than three recapture events so could not be calculated from the three-year site. Jolly-Seber models were used to estimate population size and Cormack-Jolly-Seber models were used to calculate recapture and survival probabilities.

|  | **Demographics and Survival** | | | | | **Phenotypic Traits (x̅ ± σ_x_)** | | | | | | |
| --- | --- | --- | --- | --- | --- | --- | --- | --- | --- | --- | --- | --- |
|  |  |  |  |  |  | **Dorsal Color Space** | | | **Anatomy (SVL-adjusted)** | | | |
|  | **Pop. size (N)** | **Pop. density (N/ha)** | **Sex ratio (F:M)** | **Survival prob.**  **ɸ ± σ_x_** | **Capture prob.**  **p ± σ_x_** | **(L)**  **Lightness** | **(a)**  **Green→**  **red** | **(b)**  **Blue→**  **yellow** | **Interlimb length** | **Pelvic width** | **Head Depth** | **Head Width** |
| ***S. cowlesi*** | 276 | 13.1 | 52.5:47.5 | 0.45 ± 0.55 | 0.55 ± 0.58 | 43.6 ± 12.0 | 3.1 ± 3.2 | 10.2 ± 5.6 | 0.001 ± 0.051 | -0.015 ± 0.076 | 0.002 ± 0.062 | -0.003 ± 0.055 |
| ***H. maculata*** | 208 | 6.4 | 50.5:49.5 | 0.68 ± 0.56 | 0.57 ± 0.57 | 71.6 ± 9.5 | 4.3 ± 3.6 | 17.6 ± 4.9 | 0.003 ± 0.050 | -0.033 ± 0.088 | -0.015 ± 0.051 | -0.01 ± 0.051 |

**Table 2: Within Versus Among Individual Variation in Color Analysis Results:** ANOVA output (F-statistics and R^2^ values) for within versus among individual variation (linear model of trait ~ individual) and delta Akaike Information Criteria (ΔAIC) scores from the null (no-trait) model for models of survival by color for the three elements of CIELab color space for each species. Higher F-statistics and R^2^ values indicate lower intraindividual variation and are bolded (where R^2^ > 0.5 means at least 50% of the trait variability is attributable to across individual – or intraspecific – variation), as are models where use of first, median, and last measurements all had higher support than the null no trait model (negative ΔAIC). Measurements were taken from color averaged over a 4 mm by 4 mm square on both the dorsal and dorsolateral surface of each lizard.

|  | | ***S. cowlesi*** | | | | | ***H. maculata*** | | | | |
| --- | --- | --- | --- | --- | --- | --- | --- | --- | --- | --- | --- |
|  | | **Individual Variation (ANOVA)** | | **CJS Model Comparison**  **(AIC)** | | | **Individual Variation (ANOVA)** | | **CJS Model Comparison**  **(AIC)** | | |
| **Model** | **Anatomical Trait** | **F-statistic** | **R^2^** | **First_ΔAIC_** | **Median_ΔAIC_** | **Last_ΔAIC_** | **F-statistic** | **R^2^** | **First_ΔAIC_** | **Median_ΔAIC_** | **Last_ΔAIC_** |
| **Dorsal** | **(L) lightness** | **8.2** | **0.70** | 1.98 | 0.42 | -0.88 | **4.5** | **0.53** | 0.98 | -0.84 | -1.27 |
|  | **(a) green → red** | **4.3** | **0.52** | **-0.35** | **-14.07** | **-19.64** | **10.4** | **0.76** | 1.99 | 1.54 | -1.30 |
|  | **(b) blue → yellow** | **5.3** | **0.59** | 0.68 | -2.08 | -2.87 | **4.7** | **0.55** | 0.62 | 0.42 | -1.77 |
| **Dorsolateral** | **(L) lightness** | **7.4** | **0.68** | 1.64 | 1.96 | 1.97 | 2.5 | 0.33 | 1.93 | 0.69 | 2.00 |
|  | **(a) green → red** | **4.3** | **0.52** | 0.68 | -3.34 | -5.04 | **13.4** | **0.80** | 1.96 | 1.97 | 1.11 |
|  | **(b) blue → yellow** | **4.3** | **0.52** | 1.94 | 1.92 | 1.95 | **4.5** | **0.53** | 1.47 | 1.83 | 1.81 |

**Table 3: Within Versus Among Individual Variation in Anatomy Analysis Results:** ANOVA output (F-statistics and R^2^ values) for within versus among individual variation (linear model of trait ~ individual) and delta Akaike Information Criteria (ΔAIC) scores from the null (no-trait) model for models of survival by body size (snout-vent-length) and anatomical traits adjusted for body size for each species. Higher F-statistics and R^2^ values indicate lower intraindividual variation and are bolded (where R^2^ > 0.5 means at least 50% of the trait variability is attributable to across individual – or intraspecific – variation), as are models where use of first, median, and last measurements all had higher support than the null no trait model (negative ΔAIC).

|  | | | ***S. cowlesi*** | | | | | ***H. maculata*** | | | | |
| --- | --- | --- | --- | --- | --- | --- | --- | --- | --- | --- | --- | --- |
|  | | | **Individual Variation (ANOVA)** | | **CJS Model Comparison**  **(AIC)** | | | **Individual Variation (ANOVA)** | | **CJS Model Comparison**  **(AIC)** | | |
| **Model** | | **Anatomical Trait** | **F-statistic** | **Adjusted R^2^** | **First_ΔAIC_** | **Median_ΔAIC_** | **Last_ΔAIC_** | **F-statistic** | **Adjusted R^2^** | **First_ΔAIC_** | **Median_ΔAIC_** | **Last_ΔAIC_** |
| **Body size** | | **Snout-Vent-Length** | **8.3** | **0.71** | 1.78 | -7.06 | -15.04 | 1.9 | 0.23 | **-0.69** | **-9.76** | **-49.34** |
| **SVL-adjusted** | **Body shape** | **Weight (condition)** | 2.3 | 0.29 | 1.99 | -0.14 | -4.00 | 4.0 | 0.50 | 1.97 | 1.62 | 1.73 |
|  |  | **Interlimb length** | **5.1** | **0.57** | **-0.16** | **-4.03** | **-2.30** | **4.3** | **0.52** | 1.80 | 1.32 | -0.57 |
|  |  | **Pelvic width** | 3.9 | 0.49 | -2.66 | 1.97 | 1.93 | 3.8 | 0.48 | 1.85 | -0.88 | -0.67 |
|  | **Head shape** | **Head length** | **7.0** | **0.66** | 0.11 | 0.87 | -10.60 | 3.6 | 0.46 | 2.00 | 0.45 | -4.02 |
|  |  | **Head depth** | **7.1** | **0.67** | **-1.80** | **-6.70** | **-17.52** | 3.6 | 0.46 | 1.85 | 1.74 | 1.73 |
|  |  | **Head width** | **4.1** | **0.51** | 1.75 | 2.00 | 0.81 | **5.1** | **0.58** | **-0.06** | **-1.32** | **-4.38** |
|  | **Limb length** | **Forelimb length** | **4.3** | **0.52** | 1.83 | 1.70 | -1.22 | **11.9** | **0.78** | 1.93 | 1.35 | 0.50 |
|  |  | **Hindlimb length** | 2.7 | 0.36 | 3.49 | 0.15 | 1.14 | **18.8** | **0.85** | 1.96 | 1.41 | 0.63 |
